# Primary Investigation of Low Back Pain among Saudi Arabians: A Cross-Sectional Study

**DOI:** 10.3390/ijerph191912854

**Published:** 2022-10-07

**Authors:** Asma Saad Alrushud, Dalyah Mohammed Alamam, Muneera Mohammed Almurdi, Shouq Khalid Almutairi, Razan Othman Alzahrani, Manal Salem Alanazi, Wafa Madani Dhahi, Dimah Majid Alshaiqy

**Affiliations:** Department of Health Rehabilitation Sciences, College of Applied Medical Sciences, King Saud Universit, Riyadh 11451, Saudi Arabia

**Keywords:** low back pain, patient beliefs, cross-sectional, general public, Saudi population

## Abstract

Low back pain (LBP) is a prevalent disease that affects all ages and is a symptom that induces immobility. Patients’ beliefs may influence LBP management, and adjusting detrimental beliefs is required to improve treatment outcomes. Our aim was to evaluate the prevalence of LBP within the Saudi population and beliefs regarding LBP, physical activity, rest, imaging, and medication. People with LBP were targeted with a questionnaire containing sections on demographic information and the validated Back Beliefs Questionnaire (BBQ), in addition to questions regarding imaging, physical activity, rest and medication. A total of 651 responses were received, 559 of them (86%) experienced LBP. The most common age group was those aged 18–21 (n = 221), 80% from females. The average BBQ score was 27.8 (SD = 5.58). The majority of the respondents held the following beliefs, which are contrary to the best available evidence: back pain must be rested (77.1%) and X-rays or scans are required to gain the best medical care for LBP (73.2%). The Saudi population holds unhelpful beliefs that may affect their quality of life. Healthcare professionals working with patients with LBP have an important role in changing detrimental beliefs and behaviors about the condition.

## 1. Introduction

Low back pain (LBP) is a common condition that affects people of all ages and is one of the most prevalent reasons for seeking medical care [1]. Around 80% of people suffer from LBP at some point in their lives [2], with 40% developing chronic LBP [3]. In 2019, the global age-standardized prevalence of LBP was 70 per 1000 people, and it was a major cause of the demand for rehabilitative treatment in 134 out of 204 countries [4]. In Saudi Arabia, LBP was found to be present in between 64% and 89% of the population [5].

Complications relating to LBP are mostly prompted by the underlying etiology, but they can be divided into two categories: physical and social. Physical complications include persistent pain and deformity. Social complications are usually measured by disability, including absence from work [6]. The quality of the management provided for LBP is affected by the patients’ beliefs regarding the condition and about physical activity, rest, imaging, and the use of medication [7]. All these factors can influence patients’ engagement with and adherence to a treatment plan. As a result, studying and understanding these beliefs is extremely important [8,9,10].

In the literature, patients with LBP hold some unhelpful beliefs about their situation, which may lead to the avoidance of daily activities, exercises, and work [11]. For example, patients might believe that work, activity, and exercises increase pain and that the avoidance of participation will prevent pain; this belief may result in immobility, which can end in disability [11]. Therefore, healthcare professionals have a role to play in correcting unhelpful beliefs in order to improve the quality of treatment [9,10]. They can correct their patients’ beliefs by providing appropriate strategies, such as education, motivation, resuming activities, and minimizing the associated disability [9,10].

Moreover, many people believe that physical inactivity and using medication can reduce LBP, and that radiological imaging can help with the diagnosis. The guidelines, however, demonstrate a different view [12]. A cross-sectional study highlighted that those patients who do not engage in exercises, or any physical activity, may develop unhelpful coping strategies. They are also at a higher risk of recurrence and poor outcomes of LBP [13]. According to clinical practice guidelines for the management of non-specific LBP in primary care, patients need to be advised to maintain their regular activity and must be encouraged to avoid bed rest [12]. In addition, they need to be educated and reassured that LBP is not a dangerous condition and that it has a good prognosis [12].

In addition, those guidelines recommending medication prescriptions for patients with LBP differ depending on the medication type and the duration of the symptoms [12]. Most guidelines recommend using non-steroidal anti-inflammatory drugs (NSAIDs) for a patient with acute and chronic LBP, and substitute opioids can be used if NSAIDs are not effective. Most guidelines support the use of paracetamol and acetaminophen [12]. Furthermore, anti-depressants were recommended for patients with chronic LBP when required [12]. Moreover, according to all the guidelines, routine imaging for patients with LBP is not recommended, as it incurs unnecessary costs for both the healthcare system and individuals and exposes the patient to unnecessary radiation [14]. Guidelines state that imaging should only be performed if red flags are perceptible or when the findings are expected to influence or lead to treatment [12].

Beliefs about back pain have been studied in Saudi Arabia by using the Back Beliefs Questionnaire (BBQ) to explore patients’ beliefs about back pain and its consequences [15,16]. However, the beliefs of patients regarding physical activity, rest, imaging, and medication use have never been examined in the Saudi population. Therefore, we are interested in identifying beliefs among the Saudi population regarding the prognosis and management of LBP.

Studying patients’ beliefs regarding LBP is critical [9,10], as they can affect the quality of the management provided [7], since it might help to improve the quality of treatment by providing appropriate strategies for correcting unhelpful beliefs, encouraging patients to resume activities, and minimizing the associated disability [11]. In general, this study had the aim of estimating the prevalence of LBP within the Saudi population and examining their beliefs regarding LBP, physical activity, rest, imaging, and medication use. Moreover, in this study, we hypothesize that the Saudi population holds unhelpful beliefs about the diagnosis and management options for LBP.

## 2. Materials and Methods

### 2.1. Study Design and Participants

A cross-sectional study was carried out based on an online survey targeting the Saudi population. A random sample from the Saudi population of 385 adults was the target for completed online surveys in order to estimate a prevalence rate for LBP of at least 50% with a 95% confidence interval and a 5% margin of error. The participants were recruited according to the following inclusion criteria: Saudi citizens over 18 years old, with LBP in the previous 12 months. Participants who could not complete the online survey were excluded. A text message that included a brief introduction to the study and a survey link (using Google Forms) was sent using social media, including WhatsApp and Telegram, to the public across the kingdom. Participants were initially asked if they had ever had backpain. If they answered ‘no’ then no further questions were required to be completed. If they answered ‘yes’ then subsequent questions were posed to collect information. The survey was halted after two months and the data were downloaded for analysis. This study was approved by the Institutional Review Board, College of Medicine, King Saud University in Saudi Arabia (Ref. No. 22/0118/IRB) on 3 February 2022. An online consent form was embedded with the survey.

### 2.2. Outcome Measures

A survey in Arabic about the prognosis and best management strategies of LBP was used to collect data. The original survey contained three parts. Part one concerns the participant’s demographic data (age, gender, and LBP characteristics, including pain intensity measured by a visual analog scale [VAS]). Part two consists of 14 statements about back beliefs, each item scored on a 5-point Likert scale ranging from strongly disagree (1) to strongly agree (5). Five items were used as distractors and not included in the total score (Q4, Q5, Q7, Q9, and Q11) and the remaining nine items were used for scoring (Q1, Q2, Q3, Q6, Q8, Q10, Q12, Q13, and Q14). All nine items were reversed (e.g., 5 = 1 and 2 = 4) before they were summarized to provide a total score ranging from 9 to 45. Lower scores represent more unhelpful beliefs about LBP. Part three contains six additional questions about physical activity, rest, imaging, and medication use [7]. Parts one and three of the survey [7] were translated from English into Arabic, reviewed by a peer reviewer, and piloted for clarity. Part two, consisting of the BBQ, is already translated into Arabic and the Arabic version was used [16].

### 2.3. Statistical Analysis

Data were entered in an Excel spreadsheet and checked for errors before analysis. Both the demographic and LBP characteristics of the sample were reported using descriptive statistics in terms of the number and frequency. The 95% confidence interval for prevalence has been estimated using the Clopper-Pearson exact method. The BBQ items and the six additional items on beliefs related to physical activity, rest, and the use of imaging and medication were presented as proportions trichotomized into disagreement (disagree or strongly disagree), agreement (agree or strongly agree) and being unsure (neither agree nor disagree). The total score for the BBQ was calculated according to the scoring method in Alamrani et al. [16]. Lower scores represent more unhelpful attitudes and beliefs about back pain.

## 3. Results

### 3.1. Demographic and LBP Characteristics

**Figure 1:**
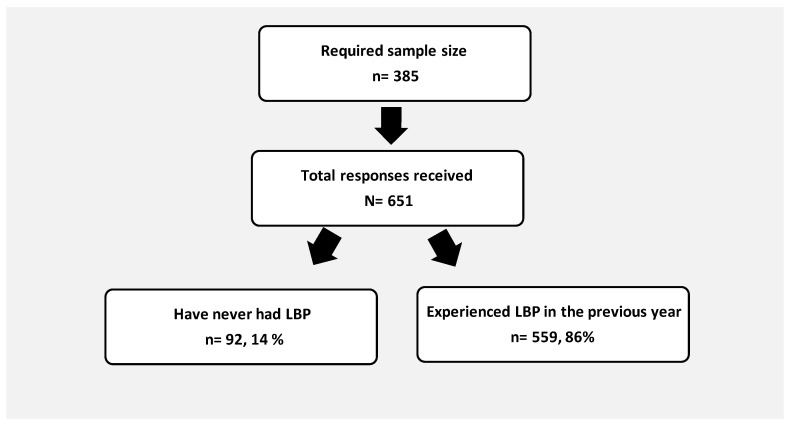
Flow diagram of the study group.

A higher-than-expected response rate occurred with the six hundred fifty-one responses to the online survey that were received. Among the replies, 92 respondents had never had LBP, resulting in a prevalence rate for LBP of 86% (95% CI = 83 to 88%), which is also statistically significantly different to the hypothesized prevalence of 50% with *p* < 0.0001. The non-LBP respondents were excluded from further analysis in this study (Figure 1). Most of the LBP respondents were female (80%), and the largest age group (39%) contained those aged 18–21 (n = 221). In terms of occupation, the highest percentage were employed (34.34%) and with respect to educational level, the highest percentage were those with a bachelor’s degree (59%). Most responses were received from the central region of Saudi Arabia (74.4%), and the cities with the most respondents to the survey were Riyadh, Tabuk, and Medina. Of those who had experienced LBP in the previous year, 314 (56.2%) reported LBP within the previous week. Regarding pain intensity across all responses, the mean was 5.4 out of 10. Most responses (61.4%) from those with LBP referred to seeking care, such as going to the hospital or taking medications when pain occurred (Table 1 and Table 2).

### 3.2. Back Beliefs Questionnaire

The mean BBQ score was 27.8 (SD = 5.58), calculated according to the scoring procedures in Alamrani et al. [16]. Approximately 20% of the respondents neither agreed nor disagreed (Neutral) with the belief statements across all nine categories. Most of the respondents had negative views about LBP that were contradictory to the evidence-based management: 1. Later in life back trouble gets progressively worse (item 14); 2. Back trouble must be rested (item 13); and 3. Back trouble means periods of pain for the rest of one’s life (item 3) (Table 3).

### 3.3. Beliefs about Physical Activity, Rest, and the Use of Imaging and Medication

With regard to beliefs about LBP, 409 (73.2%) of the participants believed that using X-rays or scans are necessary to get the best medical care for their pain and 321 (57.4%) agreed that everyone with LBP should have spine imaging. In addition, 393 (70.3%) of the participants agreed that they should rest until their back pain gets better and only 206 (36.9%) agreed that they should stay active and avoid resting. In terms of analgesic use, 172 (30.8%) agreed that using simple painkillers was enough to control most back pain. On the other hand, 252 (45%) of the participants were of the belief that most back pain settles quickly and they could do their normal activities, such as going to work (Table 4).

## 4. Discussion

This study was conducted to estimate the prevalence of LBP within the Saudi population and to examine their beliefs toward LBP, physical activity, rest, imaging, and medication use. Based on the survey responses, the prevalence could be about 86%, which is within the range (64–89%) that was estimated in a previous systematic review of the prevalence of LBP in the Saudi population [5]. However, LBP is one of the most common global health issues and is a leading cause of disability [17].

The clinical outcomes of patients can be affected by the beliefs that the individuals hold about the causes of their LBP [9]. Patients’ beliefs can affect their choice of healthcare and preference for treatment [18]. Therefore, the quality of the care provided might be improved by understanding the patients’ expectations and beliefs regarding treatment [7]. The majority of the individuals who responded to our survey believe that back pain has unhelpful consequences, which supports our hypothesis. They also hold beliefs that are counter to evidence-based practice concerning the nature, prognosis, and proper treatment of LBP. For example, over 90% of the survey participants believe (or are uncertain) that back trouble gets worse in later life and that back trouble must be rested. Furthermore, over 60% believe (or are uncertain) that back trouble means periods of pain for the rest of their life. In recent research, the BBQ was used in various countries, such as Canada, Brazil, the Caribbean, Turkey, Nigeria, France, and Australia, to investigate people’s beliefs about back pain and pain management [7,8,19,20,21,22,23]. Respondents in all the previous studies agreed, on average, that back pain has unhelpful consequences, which resulted in BBQ ratings in the range 23.2–28. Therefore, the mean BBQ we obtained (27.8) can be regarded as low compared to the maximum value of 45 [16]. Moreover, in Saudi Arabia specifically, two studies were conducted that found average BBQ scores of 25.8 [16] and 28.6 [15]. Their findings are consistent with ours that participants held unhelpful beliefs about LBP. A study conducted of physiotherapy care settings in Saudi Arabia [15] reported that most of the participants agreed with some unhelpful statements. For example: “There is no real treatment for back trouble” and “Back trouble will eventually stop you from working.” These results might indicate that the Saudi population still holds unhelpful beliefs about LBP.

The item most often agreed with in three of the previous studies was that “Later in life back trouble gets progressively worse” [7,8,19]. This is similar to the findings of this study. Even though back pain may become more disabling with age, this expectation is overly pessimistic, since most cases of back pain have a very good prognosis and back pain often follows a changeable trajectory for many years before becoming permanent. Therefore, more information on the prognosis of back pain should be provided to the public [10].

Moreover, the items “Back trouble must be rested” and “Back trouble means periods of pain for the rest of one’s life” were the most agreed upon in our study and in previous studies. The statements in these items are contrary to the evidence base about the management of LBP, so patient beliefs must be corrected about the nature of the symptom and they must be encouraged to remain active and avoid rest [12].

Furthermore, there is also a similarity between our findings and a previous study in Canada with respect to the statement that “Back trouble will eventually stop you from working,” as most of the participants agreed with the statement. This may lead to work absences due to pain resulting in activity limitation, which is highly concerning for those people from low-income countries. It is also the main reason for sick leave and absence from work when compared to any other musculoskeletal condition [1].

Management strategies of LBP in the past included the use of routine imaging, medication use, and advice to rest and avoid physical activity [24]. More recently, these management strategies were found not to be effective [25,26,27]. Clinical practice guidelines for the management of LBP over the last 20 years recommend avoiding excessive rest and promoting advice to stay active, minimizing the use of pain killers, and limiting the use of imaging to specific serious conditions, such as infection, cauda equina, cancer, and nerve root compression [12].

In terms of beliefs about imaging, using pain killers, and the prognosis of LBP, the participants of our study hold similar beliefs to the Canadian population [7]. More than 50% of the participants agreed with statements on the necessity for X-rays or scans to obtain the best medical care and that everyone with LBP should undergo imaging. Evidence shows that less than 20% of patients with LBP are over-prescribed imaging [28]. This could be attributed to the patients’ beliefs and expectations, as they believe that an image will enable the best diagnosis for their back pain [29]. In most cases of LBP, studies demonstrate that imaging is not helpful for diagnosis and may lead to a worse outcome in some cases [30]. In addition, less than 50% of our sample agreed that simple analgesia is enough to control back pain and that most cases settled quickly, which aligns with findings from the Canadian study referred to earlier [7]. Regarding rest and activity, 70.3% of the participants of our study agreed that someone with LBP should have rest until they get better, while only 23.9% in the other study agreed [7]. Another result was that about 37% of our sample agreed that someone with LBP should stay active, which contrasts with the findings from Canada [7], as 55.2% of those participants agreed with the statement and their beliefs were within an estimated range (55–65%) of public beliefs from previous studies [7,23]. However, the research demonstrates that recovery is low with too much rest, as activity should be encouraged to enhance recovery and that medication use had little effect on pain management [25,26,27].

The strength of this study lies in a sample size obtained by random sampling via an online survey that covered all regions of Saudi Arabia. However, a limitation was the inclusion of a high proportion of female participants, thus limiting the ability to investigate the contribution of gender to beliefs about LBP. The responses to the survey demonstrated that the Saudi public holds unhelpful beliefs about LBP that will have a negative effect on their treatment outcomes. Once an individual holds incorrect information that is the opposite of the clinical guidelines, this creates a significant challenge for healthcare services. Healthcare professionals must clarify the evidence base for LBP treatment to their patients and reassure them that pain can be managed and will not last for the rest of their lives. Furthermore, patients need to be advised to stay active and to avoid bed rest, in order to prevent complications. The role of healthcare professionals is essential in giving individuals full knowledge of their situation so that they can gain the optimum benefits from treatment.

## 5. Conclusions

Extrapolating from our sample, the prevalence of LBP in the Saudi population could be as high as 86%, offering the high possibility that a large number of the population will seek medical advice or treatment for LBP. Beliefs of patients regarding physical activity, rest, imaging, and medication in the Saudi population have not been examined previously. Most of the population hold unhelpful beliefs about LBP, mainly with regard to LBP diagnosis and management: for example, that they should stop working and rest, their backs will get progressively worse, and imaging is required for the best medical care. To improve treatment outcomes, healthcare professionals who work with LBP cases need to educate their patients and change their beliefs regarding LBP.

## Figures and Tables

**Table 1:** Demographic outcomes of the survey participants (n = 559).

Demographic Characteristics	Number (%)
**Age**	
18–27	221 (39.5)
28–37	101 (18.1)
38–47	98 (17.5)
48–57	97 (17.4)
58–67	37 (6.6)
68 or more	5 (0.9)
**Gender**	
Female	449 (80.3)
Male	110 (19.7)
**Educational level**	
Less than elementary	5 (0.9)
Elementary	7 (1.3)
Middle	18 (3.2)
High	100 (17.9)
Diploma	55 (9.8)
Bachelor’s	330 (59.0)
Master’s	30 (5.4)
Doctorate or higher	14 (2.5)
**Residential area**	
Central region	416 (74.4)
Western region	63 (11.3)
Eastern region	20 (3.6)
Northern region	43 (7.7)
Southern region	17 (3.0)
**Occupation**	
Student	148 (26.48)
Employee	192 (34.35)
Unemployed	154 (27.55)
Retired	65 (11.63)

**Table 2:** Low back pain characteristics of survey participants (n = 559).

Item	Yes (n, %)	No (n, %)	Prevalence (95% CI)
Have you ever had back pain? *	559 (85.9)	92 (14.1)	86% (83%, 88%)
Have you had lower back pain in the last 12 months?	519 (92.8)	49 (8.8)	
Have you had lower back pain in the past week?	314 (56.2)	245 (43.8)	
When back pain occurs, have you been taking some painkillers or looking for care such as going to the hospital?	343 (61.4)	216 (38.6)	
	**Mean**	SD **	95% CI ***
Describe the intensity of the pain you last felt in your lower back from 1 to 10 (where 1 means the pain was very mild and 10 means the pain was very severe).	5.4	2.26	5.2, 5.9

* Total N = 651; all other items: N = 559; n = number of respondents; ** SD = standard deviation; *** CI = confidence interval.

**Table 3:** Back Beliefs Questionnaire (9 items for scoring) (n = 559).

BBQ Item	Disagree (1 and 2) n (%)	Neutral (3) n (%)	Agree (4 and 5) n (%)	Mean Score (SD) 95% CI
1. There is no real treatment for back trouble (item 1)	267 (47.8)	172 (30.8)	120 (21.5)	3.40 (1.044) 3.32, 3.49
2. Back trouble will eventually stop you from working (item 2)	238 (42.6)	124 (22.2)	197 (35.2)	3.10 (1.127) 3.01, 3.19
3. Back trouble means periods of pain for the rest of one’s life (item 3)	209 (37.4)	123 (22)	227 (40.6)	2.98 (1.096) 2.89, 3.07
4. Back trouble means everything in life is worse (item 6)	298 (53.3)	106 (19)	155 (27.7)	3.36 (1.157) 3.27, 3.46
5. Back trouble might mean you end up in a wheelchair (item 8)	426 (76.2)	83 (14.8)	49 (8.8)	4.03 (0.973) 3.95, 4.11
6. Back trouble means long periods of time off work (item 10)	266 (47.6)	143 (25.6)	150 (26.8)	3.24 (1.008) 3.15, 3.32
7. Once you have had back trouble there is always a weakness (item 12)	331 (59.2)	88 (15.7)	140 (25)	3.45 (1.115) 3.36, 3.54
8. Back trouble must be rested (item 13)	56 (10)	72 (12.9)	431 (77.1)	2.10 (0.887) 2.03, 2.17
9. Later in life back trouble gets progressively worse (item 14)	51 (9.1)	103 (18.4)	405 (72.4)	2.19 (0.846) 2.12, 2.27
**Total score**				27.86 (5.580) 27.40, 28.32

**Table 4:** Participants’ beliefs about activity, rest, and the use of imaging and medication (n = 559).

Belief Statement	Disagree (1 and 2) n (%)	Neutral (3) n (%)	Agree (4 and 5) n (%)
1. X-rays or scans are necessary to get the best medical care for low back pain	42 (7.5)	108 (19.3)	409 (73.2)
2. Everyone with low back pain should have spine imaging (e.g., X-ray, CT, MRI)	111 (19.8)	127 (22.7)	321 (57.4)
3. If you have back pain, you should rest until it gets better	60 (10.7)	106 (19)	393 (70.3)
4. If you have back pain, you should try to stay active (e.g., performing normal daily activities before feeling pain)	191 (34.2)	162 (29)	206 (36.9)
5. Simple painkillers are usually enough to control most back pain	210 (37.6)	177 (31.7)	172 (30.8)
6. Most back pain settles quickly, and you can get on with normal activities such as going to work	150 (26.8)	157 (28.1)	252 (45.1)

CT = computer tomography; MRI = magnetic resonance imaging.

## Data Availability

The data used in this study and that support the findings are available from the corresponding author on reasonable request, but are not available to the public due to privacy or ethical restrictions.
